# Synergistic Effects of Proprioceptive Neuromuscular Facilitation and Manual Lymphatic Drainage in Patients with Mastectomy-Related Lymphedema

**DOI:** 10.3389/fphys.2017.00959

**Published:** 2017-11-28

**Authors:** Kyung-Jin Ha, Sang-Yeol Lee, Hojun Lee, Seung-Jun Choi

**Affiliations:** ^1^Department of Sports and Health Science, Kyungsung University, Busan, South Korea; ^2^Department of Physical Therapy, Kyungsung University, Busan, South Korea; ^3^Korea Proprioceptive Neuromuscular Facilitation Association, Busan, South Korea; ^4^Department of Rehabilitation Medicine, College of Medicine, Seoul National University Bundang Hospital, Seongnam, South Korea

**Keywords:** proprioceptive neuromuscular facilitation, manual lymphatic drainage, lymphedema, breast cancer, mastectomy

## Abstract

**Purpose:** Manual lymphatic drainage (MLD) and proprioceptive neuromuscular facilitation (PNF) are potential therapeutic strategies to reduce mastectomy-induced edema. The purpose of this study was to investigate whether the combination of these therapies would induce synergistic effects to treat lymphedema-related complications and to analyze a possible physiological mechanism involved in the observed effects.

**Methods:** A total of 55 patients diagnosed with mastectomy-induced lymphedema were recruited and randomized into three experimental groups: PNF group (*n* = 17), MLD group (*n* = 20), and PNF + MLD group (*n* = 18). They were subjected to designated rehabilitation program three times a week for 16 weeks. ROM (flexion of the shoulder joint), edema size, arterial blood flow velocity, and degree of pain and depression were measured every 4 weeks over experimental period.

**Results:** Lymphedema volume, VAS pain scale, and Beck depression scale were decreased in PNF and MLD groups for 16 weeks in a time-dependent manner. In combination, a greater reduction of these variables was observed over 16 weeks compared to each PNF and MLD. While axillary arterial blood circulation rate in the affected extremity was increased in both PNF and PNF + MLD groups over 16 weeks, this value was not increased in MLD group throughout the experimental period. A greater reduction of scales of VAS pain and Beck Depression Inventory (BDI) was observed in PNF + MLD group after the 16 week-treatment, as compared to each PNF and MLD group. Pearson's coefficients test demonstrated that there are significant correlation of depression against pain (*r* = 0.616, *p* < 0.01), ROM (*r* = −0.478, *p* < 0.01), and lymphedema size (*r* = 0.492, *p* < 0.01).

**Conclusion:** The combination of MLD and PNF induces potent synergistic effects on edema volume, shoulder range of motion (ROM), pain, and depression in patients with lymphedema. In addition, an increased rate of axillary arterial blood flow in PNF-treated patients provide a potential physiological mechanism by which local arterial pulsation in the affected extremity plays a positive role in the treatment of lymphedema. Therefore, it is suggested to incorporate an element of PNF into traditional MLD method to facilitate treatment process for patients with lymphedema.

## Introduction

Lymphedema is a pathological state of chronic tissue swelling induced by the restriction of lymph drainage (Armer, 2005). It is reported that more than 20% of breast cancer patients are inflicted with lymphedema (Ezzo et al., 2015). Since most of the current surgical procedures are accompanied by removal of lymph nodes in unilateral or bilateral upper extremities (Fairey et al., 2002), the etiology of lymphedema is primarily caused by reduced capacity of processing lymph fluid in axillary region of the affected area (Bassler, 1996). Although lymphedema is not a life-threatening disorder, this chronic condition can cause not only physical discomfort, but also functional impairment, ultimately leading to anxiety and depression (Ridner, 2009; Ha and Choi, 2014). Moreover, accumulating evidences proved that lymphedema is tightly coupled with the development of severe pathological complications such as lymphangitis and cellulitis (Pereira de Godoy et al., 2009; Park et al., 2016). Thus, an effective strategy for lymphedema is warranted. With the advent of microsurgery, the application of multiple lymphatic-venous anastomoses has become a common surgical treatment for lymphedema (Campisi and Boccardo, 2004; Campisi et al., 2006). However, due to lack of convincing evidence of the success, a significant portion of patients with lymphedema opt for non-surgical therapeutic treatments (Damstra et al., 2009). Among them, manual lymphatic drainage (MLD) has garnered attention as a non-surgical treatment. MLD is defined as a manual massage technique which is applied on skin surface along with anatomical lymphatic pathways to promote drainage from the affected extremity (Huang et al., 2013). At present, the application of MLD combined with other supplemental methods, such as complex decongestive physiotherapy is regarded as the most effective non-surgical therapeutic method. These treatments commonly cause an increase in the flow of lymph, the contraction rate of lymphatic vessels, and the re-absorption of proteins into lymphatic vessels, leading to transferring the fluid of edema in the direction of lymph nodes (Williams et al., 2002; Moseley et al., 2007; Williams, 2010; Ezzo et al., 2015).

The development of lymphedema is tightly associated with reduction in muscle flexibility and joint movements (Thomas-Maclean et al., 2008). Moreover, more aggressive breast cancer treatment involving mastectomy induces a greater restriction in shoulder range of motion (ROM) in the affected arm (Smoot et al., 2010). Since deficits in ROM may interfere with daily activities, leading to a diminished quality of life, it is recommended that patients require vigilant musculoskeletal attention following mastectomy to restore normal range of shoulder elevation.

Proprioceptive neuromuscular facilitation (PNF) is a stretching technique mainly utilized to improve ROM in the field of sports medicine. A key element of PNF technique induces joint movements within a functional range in the absence of pain (Sharman et al., 2006). PNF has been well-proven to improve flexibility and muscular strength, and vascular blood flow (Feland and Marin, 2004). Since contraction of skeletal muscles propels lymph flow in the direction to cardiovascular system (Drewes et al., 2007), it is hypothesized that PNF-induced muscle movement has positive role in the reduction of lymphedema. Although a couple of studies suggested potential of PNF's therapeutic effects in patients with lymphedema (Hwang et al., 2013; Ha and Choi, 2014), synergistic effects of the two prevalent manual methodologies, PNF and MLD, have not been investigated. Therefore, the main objectives of the study are two-fold: to investigate if the combined treatment of PNF and MLD would improve lymphedema-related complications and to study a possible physiological mechanism involved in the observed effects.

## Methods

### Subject

The subjects consisted of 55 female patients who had been diagnosed with breast cancer and underwent mastectomy. Their physical characteristics are shown in Table 1. The criterion for entry in the study was a minimum of 2 cm difference in circumference between both arms (Andersen et al., 2000). Patients with multiple lymphedema sites were excluded from the study. All the participants were provided sufficient information on the test's purpose, procedures, and risks, and then submitted a written consent. To minimize extra variables in the subjects, Stretch compression bandages (Lohmann & Rauscher, USA) and lymph pads (Mediven, USA) were treated to all the subjects for 46 h every 2 days in a supportive and time-controlled manner over the course of the study. All the dependent variables were measured 30 min after bandage removal. They were also recommended to inhibit programmed exercise, drinking, smoking, and additional medication. This study was carried out in accordance with the protocol of Ethical Committee at Kyungsung University (KSU14-05-001).

**Table 1:** Physical characteristics of the participants.

**Group**	**Age**	**Height (cm)**	**Weight (kg)**	**BMI (kg/m^2^)**
PNF (*n* = 17)	50.76 ± 0.94	159.54 ± 0.79	63.41 ± 1.17	24.92 ± 0.46
MLD (*n* = 20)	54.05 ± 0.88	159.63 ± 0.85	61.59 ± 0.98	24.21 ± 0.42
PNF + MLD (*n* = 18)	53.39 ± 0.83	158.70 ± 0.75	65.96 ± 1.56	26.21 ± 0.63

### Study design

**Figure 1:**
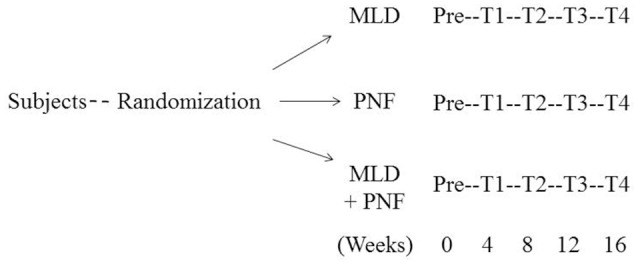
Schematic diagram of study design.

Patients diagnosed with upper-extremity lymphedema were divided into three groups: PNF rehabilitation group (*n* = 17), MLD treatment group (*n* = 20), and PNF + MLD treatment group (*n* = 18). They were subjected to respective rehabilitation program three times a week for 16 weeks. Each session consisted of 30 min per session. PNF techniques consisted of rhythmic initiation (RI) and a combination of isotonic, contract-relax, and hold-relax. Both PNF and MLD were performed by a certified technician. ROM (flexion of the shoulder joint), edema size, blood flow velocity, degree of pain, and psychological variables were measured every 4 weeks until the end of 16-week program in a time-dependent manner (Figure 1).

### Measurement and analysis methods

#### Range of motion (ROM)

The ROM in the shoulder joint was measured using a goniometer, according to the instruction released by the Korean Agency for Technology and Standards (KATS), to measure flexion in the shoulder joint in the affected arm. With patients in the supine position, the angle at the point of limitation of shoulder joint movement was evaluated. The flexion was measured as the angle of the affected arm elevated vertically from position in which the body was fixed.

#### Edema size

Edema in the upper extremity was measured using the Arm Circumference Gauge (Sammons Preston, USA). Edema size was measured at both sides of 10 cm proximal and distal to the ulnar olecranon. The values were obtained by comparing the circumferences of the affected arm and the non-affected arm. They were obtained every 4 weeks until the end of 16-week program. Data were presented using the following formula. Rate of edema (% excess) = [(affected side − non-affected side)/non-affected side] × 100.

#### Blood flow velocity

The blood flow velocity was measured in the affected rotator cuff using an ultrasonic Doppler rheometer (ES-1000SP II, Hedeco, Japan). In detail, the maximum blood flow velocity during the contraction phase, the maximum blood flow velocity during the relaxation period, and the average blood flow velocity were measured every 4 weeks until the end of 16-week program. The data were presented as ml/s.

#### Pain and depression

The degree of pain was used as an indirect indicator of muscle activation. The degree of pain was measured based on the visual analog scale (VAS), which is widely used in clinical settings to quantify pain severity (Kelly, 2001). The level of pain was examined during the ROM measurement of shoulder joints. The subjects were instructed to check a straight line graph once a month, which displays gradation from “no pain” = 0 to “unbearable pain” = 10. The values were obtained every 4 weeks until the end of 16-week program. Depression levels of the subjects were evaluated using the Beck Depression Inventory (BDI), developed by Beck et al. (1961). There are 21 categories; each of which has four options. Participants were instructed to select the suitable option reflecting their current emotional status. The values were obtained every 4 weeks until the end of 16-week program.

### Statistics

Descriptive statistical analysis was performed to present the general characteristics of the study subjects. Data were presented as mean ± SEM. Shapiro-Wilk and Mauchly's test were performed to confirm normality and sphericity of the data (*P* > 0.05). And repeated measure ANOVA and Tukey-Kramer *post-hoc* test were used to examine the difference in measured variables among groups in a time-dependent fashion. Pearson's correlation coefficients were tested to examine the relationship between depression and other lymphedema-related symptoms. The statistical significance level was set at *p* < 0.05. All the statistical analyses were performed using SPSS Version 18.

## Results

### Lymphedema volume (cm)

**Figure 2:**
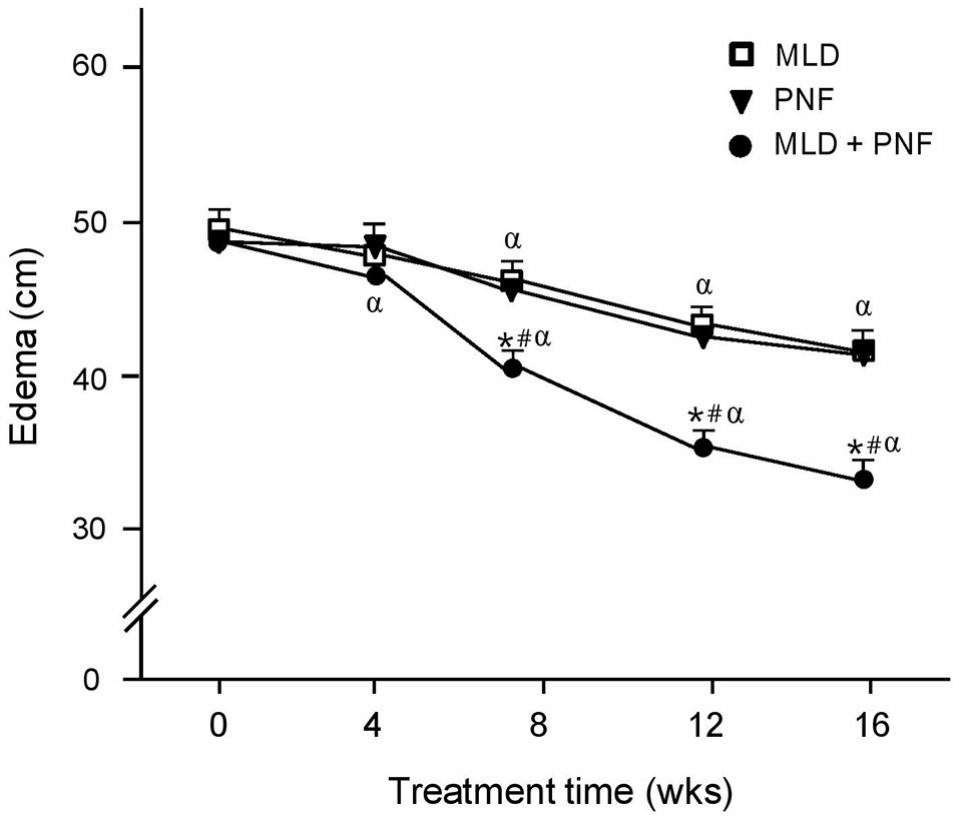
The effect of MLD, PNF, and MLD + PNF on lymphedema size over 16 weeks. Lymphedema size in the affected extremity was measured every 4 week until the end of experiment. ^*^p < 0.05; MLD vs. MLD + PNF, ^#^*p* < 0.05; PNF vs. MLD + PNF, and ^α^*p* < 0.05 vs. Wk 0.

The upper extremity lymphedema was decreased gradually in all the groups over the 16 week-treatment period (Figure 2). Specifically, as compared to week 0, a significant and immediate reduction in lymphedema size was observed in the PNF + MLD group after a 4 week-treatment and it decreased up to 26% after 16 weeks (*p* < 0.01). In the case of the PNF and MLD groups, lymphedema volume in both groups began to decrease 8 weeks later, and continually decreased up to 16 and 13% after 16 weeks as compared to pre-treatment (*p* < 0.01). The group differences were observed between PNF + MLD and other treatment groups (MLD and PNF) at 8, 12, 16 weeks (*p* < 0.01), suggesting that the combined treatment induces a potent synergistic effect on reduction of lymphedema.

### Blood circulation velocity (ml/s)

**Figure 3:**
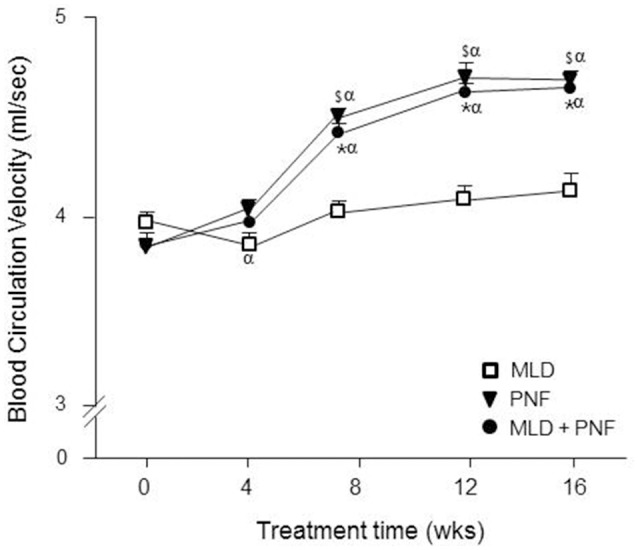
The effect of MLD, PNF, and MLD + PNF on arterial blood circulation rate over 16 weeks. Axillary arterial blood circulation rate was measured in the affected extremity every 4 week until the end of experiment. The data were expressed as ml/sec. ^*^p < 0.05; MLD vs. MLD + PNF, ^$^*p* < 0.05; MLD vs. PNF, and ^α^*p* < 0.05 vs. Wk 0.

As compared to week 0, the blood circulation velocity was increased gradually in PNF + MLD and PNF groups after the 16 week-treatment (*p* < 0.01) (Figure 3). MLD did not induce a significant change in blood circulation velocity after 16 weeks. In detail, a significant increase in blood circulation velocity was observed after 8 weeks in both PNF + MLD and PNF groups (*p* < 0.01) and it continually increased up to 20.4 and 21.3%, respectively until the end of experiment (*p* < 0.01). A non-significant increase (2.8%) was observed in MLD group after the 16 week-treatment. The group difference was observed between MLD and other groups (PNF and PNF + MLD) at 8, 12, 16 weeks (*p* < 0.01), suggesting that axillary blood circulation velocity is increased by PNF treatment in the affected extremity, but not by MLD treatment.

### Shoulder ROM

**Figure 4:**
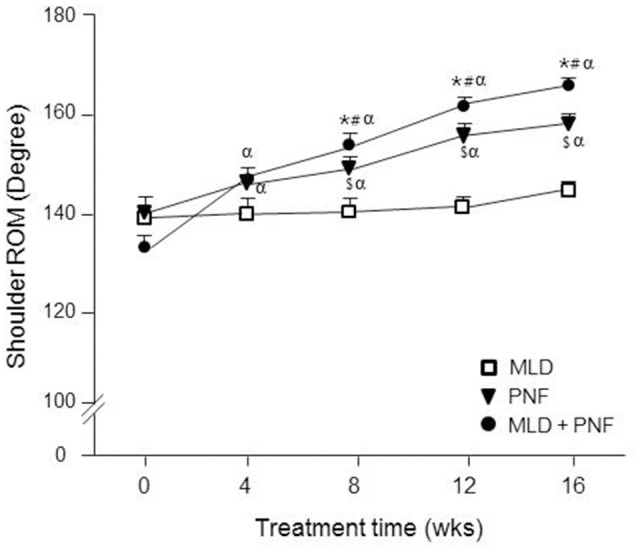
The effect of MLD, PNF, and MLD + PNF on shoulder ROM over 16 weeks. Shoulder ROM was measured in the affected extremity every 4 week until the end of experiment. ^*^p < 0.05; MLD vs. MLD + PNF, ^#^*p* < 0.05; PNF vs. MLD + PNF, ^$^*p* < 0.05; MLD vs. PNF, and ^α^*p* < 0.05 vs. Wk 0.

The ROM in shoulder joint flexion was increased gradually in PNF + MLD and PNF group while no change was observed over the treatment period in MLD group (Figure 4). Specifically, as compared to week 0, a significant increase in the ROM was observed in the PNF + MLD and PNF groups after 4 weeks (*p* < 0.05) and it gradually increased up to 25 and 13%, respectively (*p* < 0.01). A non-significant increase (4%) was observed in MLD group after the 16 week-treatment. The group differences were observed among all the groups tested at 8, 12, 16 weeks (PNF vs. MLD: *p* < 0.01, PNF vs. PNF + MLD: *p* < 0.05, MLD vs. PNF + MLD: *p* < 0.01), showing that PNF + MLD treatment has the greatest effect, followed by PNF and MLD.

### VAS pain scale

**Figure 5:**
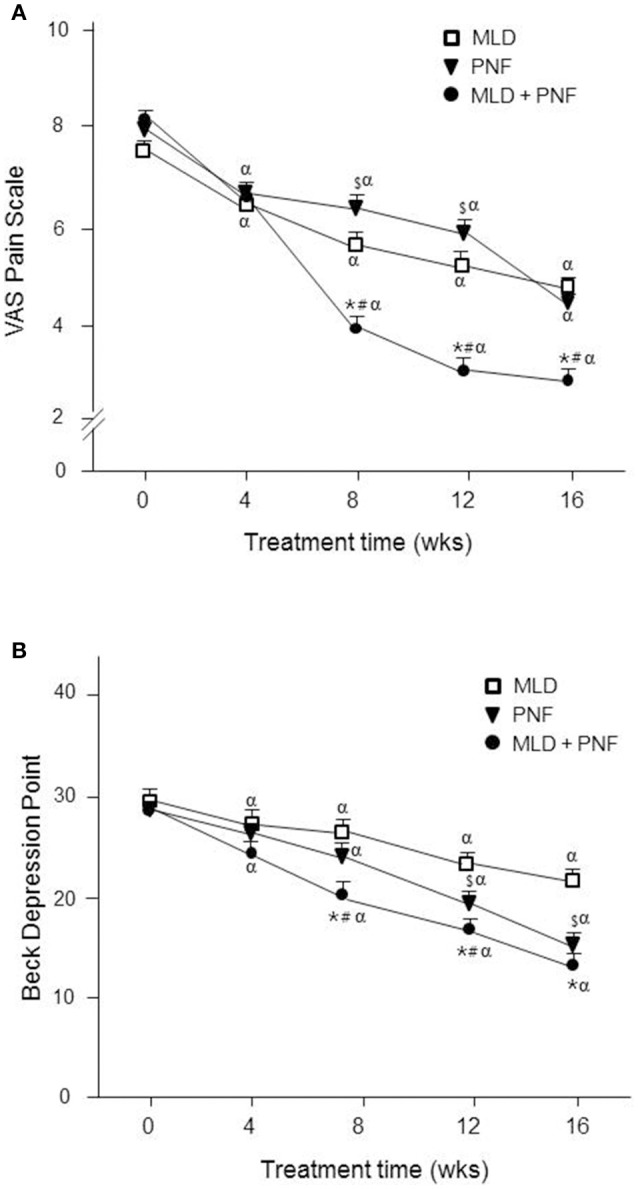
The effect of MLD, PNF, and MLD + PNF on pain and depression over 16 weeks. **(A)** VAS pain scale and **(B)** Beck depression point were measured every 4 week until the end of experiment. ^*^p < 0.05; MLD vs. MLD + PNF, ^#^*p* < 0.05; PNF vs. MLD + PNF, ^$^*p* < 0.05; MLD vs. PNF, and ^α^*p* < 0.05 vs. Wk 0.

As compared to week 0, VAS pain scale was decreased gradually in all the groups over the 16 week-treatment (Figure 5A). Specifically, the greatest reduction was observed after 16 weeks in PNF + MLD group (71%, *p* < 0.01), followed by PNF (49.6%, *p* < 0.01) and MLD (40.6%, *p* < 0.01), suggesting that the combined treatment is the most effective in reducing lymphedema-induced pain. The group difference was observed between PNF + MLD and other groups (PNF and MLD) after 16 weeks (*p* < 0.01), suggesting a potent synergistic effect of the combined treatment on the reduction of pain in the affected extremity.

### Beck depression inventory

As compared to week 0, BDI scale was decreased gradually in all the groups over the 16 week-treatment (Figure 5B). Specifically, the greatest reduction was observed after 16 weeks in PNF+MLD group (49%, *p* < 0.01), followed by PNF (42.6%, *p* < 0.01) and MLD (24.6%, *p* < 0.01), suggesting that the combined treatment is the most effective in reducing lymphedema-induced pain.

### Correlations of depression against lymphedema-related complications

The correlation coefficients of depression against pain, ROM, and lymphedema size are presented in Table 2. While depression was positively correlated with pain (*r* = 0.616, *p* < 0.01) and lymphedema size (*r* = 0.492, *p* < 0.01), depression was negatively correlated with ROM (*r* = −0.478, *p* < 0.01).

**Table 2:** Pearson's correlation coefficients between depression and lymphedema-related complications.

	**Pain**		**ROM**		**Edema size**	
Depression	*r*	*p*	*r*	*P*	*r*	*p*
	0.616	<0.01	−0.487	<0.01	0.492	<0.01

## Discussion

Concern over rehabilitation treatment after mastectomy has been discussed as a critical medical issue. Due to a limited success rate of surgical procedure, non-surgical therapies have garnered attention on upper lymphedema following mastectomy (Garza et al., 2017). It is regarded that combination of several non-surgical therapies plays an important role in the management of lymphedema (Moattari et al., 2013). Among them, MLD has been widely implemented in the field. Although a couple of studies suggested that PNF could be also implemented as a potential therapeutic method (Hwang et al., 2013; Ha and Choi, 2014), synergistic effects of MLD and PNF have not been explored. Here, we demonstrated for the first time that patients receiving combined MLD/PNF treatment showed a greater reduction in lymphedema symptoms over 16 weeks, compared to those receiving either treatment respectively.

Traditionally, patients with lymphedema have been recommended not to engage in repetitive exercise (Harris and Niesen-Vertommen, 2000). This conservative clinical guideline indirectly discourages patients from using their extremities in the affected side. Such limitation of regular activity is hypothesized to retard rehabilitation process, inducing atrophy, adiposity, and decreased ROM in a vicious cycle manner (Schmitz, 2010). However, the consensus throughout recent studies indicates that various types of exercise involving the affected extremity are not associated with the exacerbation of lymphedema (Harris and Niesen-Vertommen, 2000; McKenzie and Kalda, 2003; Courneya et al., 2007; Schmitz et al., 2009). In addition, a study reported that exercise intervention induces therapeutic effects on lymphedema symptoms (Schmitz et al., 2009). Despite the positive benefits of exercise, patients are hesitant to participate in repetitive exercise requiring a wide ROM mainly due to fears of movement-induced pain. Meanwhile, PNF requires patients to move the affected extremity within their ability in a designated direction in the absence of unbearable pain (Hwang et al., 2013), suggesting a possibility that traditional exercise intervention could be substituted with PNF treatment for patients with lymphedema.

In the study, when patients were treated with a course of intensive MLD or PNF respectively, swelling in the affected extremity was decreased in a similar fashion over the 16 week-treatment (Figure 2). Interestingly, when PNF was added to a course of MLD, a greater reduction of lymphedema volume was observed (Figure 2), showing physiological evidence that the combined treatment of MLD and PNF induces a strong synergistic effect on reduction of lymphedema. The observed synergistic effect implies a possibility that these two manual methods modulate the therapeutic process through different physiological mechanisms. This idea is confirmed by our result that axillary artery blood circulation velocity was increased in PNF group while this value was not increased in MLD group throughout the experimental period (Figure 3). Under resting condition, lymph flow is known to be mainly regulated by rhythmic contractions of smooth muscle walls of lymphangion under the control of autonomic nervous system (von der Weid and Zawieja, 2004). However, a study suggested that contraction of skeletal muscle adjacent to lymphatic vessels is also involved in dynamics of lymphatic flow (Havas et al., 1997). In the aspect of techniques, MLD uses light touch to move lymph out of the tissues and back into the lymphatic vessels (Moseley et al., 2007). This suggests that lymph flow is induced by direct touch of therapist. In the meanwhile, PNF elicits repetitive cycles of muscle contraction and relaxation, which pumps the flow of lymph into the lymphatic vessels and cardiovascular system indirectly (Drewes et al., 2007). Furthermore, local arterial pulsation in the affected extremity is positively related with lymphatic flow (Ezzo et al., 2015). Therefore, the accelerated reduction of edema in patients receiving the combined treatment might be explained by additional effects of PNF-induced local skeletal muscle contraction and arterial pulsation.

It has been well-documented that patients with lymphedema have reduced ROM in the affected extremity (Moattari et al., 2013). In the study, although MLD-mediated reduction of edema was observed over 16 weeks, shoulder ROM was not improved statistically over 16 weeks (Figure 4). Moreover, a greater ROM improvement was observed in PNF and MLD + PNF groups compared to MLD group. These results implicate that MLD-mediated reduction of edema alone is not sufficient to increase patients' ROM, suggesting that lymphadema patients require vigilant musculoskeletal attention following mastectomy to restore normal range of shoulder elevation. In contrast to our results, multiple studies demonstrated a possibility that MLD has a potential to increase shoulder ROM in patients with lymphedema (Moattari et al., 2012, 2013). However, previous studies acquired positive results by applying a series of programmed treatments such as compression pumping, remedial exercises, compression bandage, education, and MLD (Moattari et al., 2012, 2013). This integrative approach makes it difficult to find specific physiological mechanism of a certain therapy. In our study, MLD and/or PNF were applied to all the patients while compression bandage was treated in a time-controlled manner. Therefore, it is considered that methodological difference induced the discrepant results among studies.

PNF has been proven to enhance flexibility in patients with musculoskeletal dysfunction (Feland and Marin, 2004; Hindle et al., 2012; Lee, 2015). However, research on its application to patients with lymphedema has been extremely limited. To the best of our knowledge, the current study is the first to report that MLD combined with PNF induces potent synergistic effects to reduce lymphedema volume as well as to increase shoulder ROM in the affected extremity. Therefore, it is recommended to incorporate an element of PNF into traditional Complete Decongestive Therapy (CDT) to facilitate treatment process for patients with lymphedema.

There is a strong link between chronic pain and depression (Ohayon and Schatzberg, 2003). It was reported that chronic physical pain is a major contributor to the development of depression (Corruble and Guelfi, 2000). Pain is regarded as a critical complication of lymphedema, causing a great deal of distress and depression. In addition, the prolonged pain induces fears of recurrence and intrusive thoughts about breast cancer in a vicious cycle fashion (Passik and McDonald, 1998). Therefore, it is considered that proper management of pain is a major priority to prevent the development of depression in patients with lymphedema. In our study, the scales of pain and depression were decreased by each treatment gradually over 16 weeks (Figures 5A,B). In addition, the greatest reduction in the scale of pain and depression was observed in patients receiving the combined treatment over the experimental period. Since physical disfigurement, discomfort, and severe pain of the affected limb are primary causes of the depression (Passik and McDonald, 1998), it is perceived that the combined treatment-induced improvement of these factors contributes to a greater reduction of depression scale. This idea is further supported by significant correlation coefficients of depression against pain (*r* = 0.616, *p* < 0.01), ROM (*r* = −0.478, *p* < 0.01), and lymphedema size (*r* = 0.492, *p* < 0.01) (Table 2). This study was conducted with lymphedema patients who desired to participate in at least one of therapeutic treatments. Therefore, there is a slight limitation in the interpretation of the results due to lack of control group. However, our study is unique in that the recruited patients participated in the respective program over an extended period (16 weeks) and all the values were measured in a time-dependent manner. In future endeavor, a greater understanding of the combined effect of potential therapeutic treatments would provide new perspective into the development of promising non-surgical strategy for patients affected by mastectomy-induced lymphedema.

## Conclusion

As presented in Table 3, our time-dependent study demonstrated that the combination of MLD and PNF induces positive synergistic effects on lymphedema volume, shoulder ROM, pain, and depression in patients with lymphedema. Especially, the increased rate of axillary arterial blood flow in PNF-treated patients provide insight into a potential physiological mechanism by which local arterial pulsation in the affected extremity plays an additional role to alleviate lymphedema symptoms. To gain additional insight, future studies are warranted to evaluate the direct causality among axillary local arterial pulsation, skeletal muscle contraction, and lymphatic flow to find efficient therapeutic strategies.

**Table 3:** Summary of synergistic effects of PNF and MLD.

	**Edema**	**Abv**.	**ROM**	**Pain**	**Depression**
PNF	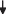	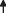	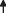	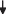	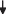
MLD	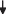	–	–	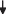	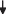
PNF + MLD	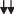	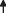	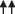	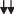	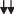

*Abv, Axillary blood velocity; ROM, range of motion. Arrow number indicates the magnitude of treatment-induced effects*.

## Author contributions

All authors listed have made a substantial, direct and intellectual contribution to the work, and approved it for publication. K-JH: acquisition and analysis of data for the work, drafting the work. S-YL: analysis and interpretation of data for the work; HL: design of the work, interpretation of data for the work, revising the work critically, final approval of the version to be published; S-JC: conception of the work, interpretation of data for the work, revising the work critically, final approval of the version to be published.

### Conflict of interest statement

The authors declare that the research was conducted in the absence of any commercial or financial relationships that could be construed as a potential conflict of interest. The reviewer AR and handling Editor declared their shared affiliation.
